# Formation of Teeth

**Published:** 1907-02

**Authors:** W. L. De LaPelriere

**Affiliations:** Winder, Ga.


### FORMATION OF TEETH

By Dr. W. L. De LaPelriere, Winder, Ga.

Another year has gone and with it many a happy dream. Again we have the pleasure of appearing before this Dental Society. The condor stoops to rest at nightfall upon the crags of the Alps, but time knows not the weight of weariness nor inclines its eyes to sleep. Thus, we are here, and I hope that the year has showered upon you many a blessing, and beg to state with all humility that I have experienced some of the sweets and some of the bitter, too—the death of my mother. On account of this, and a heavy office practice, I beg to be brief in what I shall say.

The teeth are a formation of the dermis and epidermis and are not properly a part of the skeleton, although they are found most frequently with it. They belong to the tegumentary system, which, speaking generally of animals, includes teeth, nails, horns, scales and hairs.

In all mammals teeth are made on the same general plans and similarly socketed. There is the living pulp on the inside provided with nerves and blood vessels; this is surrounded by dentine which is covered above the jaws by enamel, the hardest substance in the human body. So, in deep sea dredging teeth are found where all traces of the frame to which they belong have disappeared. Thus they are important to students of fossil life. They have been of great service in tracing the evolution of animal life during geological periods. In the herb-eating animals there have been slow changes in the pattern of the crown of the molar teeth, which can be read by students like hieroglyphics. The teeth, then, in these latter days, have resurrected the past, illuminated the dawn of existence, made it possible for the hidden to b erevealed, the dead past to live again.

No less vitally are the teeth connected with present beauty, life and happiness. How their appearance may mar or beautify the individual or be a savor of life unto life or death unto death. The American people pushing hurriedly through the crowded streets are called dyspeptics. This affects the teeth and in turn the teeth affect the whole system.

Brethren, dentistry is a philanthropic profession, and it is the duty of every honest man to regard it so and work accordingly.

I wish I could live the complete day of all existence, the dawn, noon and even-tide. Could we but have known the life of the dawn geological epochs ago! Yes, the archæologist reads it in fossil remains. Could we but live geological epochs to come and see the student of zoology as he takes up a gold-filled tooth or gold crown, and after days of study, toil and examination he comes to the conclusion that it belonged to some far-away extinct form, known probably as the gold-bug. Seriously, the dentist is here and here to stay. His services to humanity is being more thoroughly appreciated by an enlightened constituency.

In conclusion, I would like to commend the authorities of our common school system for placing in the curriculum physiology, which has a chapter in it on the proper care of the teeth. The whole subject shows the relations also of all the organisms of the body. We may expect great things in our profession in the future if we shall have a high standard of ethics between individuals, a strict law for license and one that will carry with it its face value in any state in the Union. Members of the Association, I thank you, and wish you a twelve-month's crowded happiness.

